# Nomograms for predicting the likelihood of non-sentinel lymph node metastases in breast cancer patients with a positive sentinel node biopsy

**DOI:** 10.1097/MD.0000000000018522

**Published:** 2019-12-27

**Authors:** Lihua Zheng, Feng Liu, Shuo Zhang, Yaheng Zhao, Yunjiang Liu

**Affiliations:** a Department of General Surgery; b Department of Vascular Surgery, the First Hospital of Hebei Medical University; c Department of Breast Surgery, the Fourth Hospital of Hebei Medical University, Hebei Shijiazhuang, China.

**Keywords:** breast cancer, nomogram, non-sln metastases, prediction, sentinel node

## Abstract

**Background:**

Breast cancer patients with sentinel lymph node (SLN) metastases may have a low risk of non-SLN metastases. Accurate estimates of the likelihood of additional disease in the non-SLN metastases can avoid many complications mentioned the axillary lymph node dissection (ALND). This study aims to develop a new model based on Chinese real-world patients to ascertain the likelihood of non-SLN metastases in a breast cancer patient with disease-positive SLN, enabling the surgeons to make a better choice of surgical procedures.

**Methods:**

Out of the 470 patients from CSCO Breast Cancer Database collaborated Group, a proportion of 3 (347 cases): 1 (123 cases) was considered for assigning patients to training and validation groups, respectively. Two training models were created to predict the likelihood of having additional, non-SLN metastases in an individual patient. Training model 1 was created with pathological size of the tumor, pathological type, lymphovascular invasion, the number of positive SLNs/number of total SLNs ratio, and the Her-2 status based on multivariable logistic regression (*P* < .05). Training model 2 was based on the variables in model 1 and age, estrogen receptor status, progesterone receptor status, Ki-67 count, menopause status.

**Results:**

The area under the receiver operating characteristic (ROC) curve of the training model 1 was 0.754, while the area of training model 2 was 0.766. There was no difference between model 1 and model 2 regarding the ROC curve, *P* = .243. Next, the validation cohort (n = 123) was developed to confirm the model 1's performance and the ROC curve was 0.703. The nomogram achieved good concordance indexes of 0.754 (95% CI, 0.702–0.807) and 0.703 (95% CI, 0.609–0.796) in predicting the non-SLN metastases in the training and validation cohorts, respectively, with well-fitted calibration curves. The positive and negative predictive values of the nomogram were calculated, resulting in positive values of 59.3% and 48.6% and negative predictive values of 79.7% and 83.0% for the training and validation cohorts, respectively.

**Conclusion:**

We developed 2 models that used information commonly available to the surgeon to calculate the likelihood of having non-SLN metastases in an individual patient. The numbers of variables in model 1 were less than in model 2, while model 1 had similar results as model 2 in calculating the likelihood of having non-SLN metastases in an individual patient. Model 1 was more user-friendly nomogram than model 2. Using model 1, the risk for an individual patient having ALND could be determined, which would lead to a rational therapeutic choice.

## 1 Introduction

Proponents of the performance of complete axillary lymph node dissection (ALND) after positive sentinel lymph node (SLN) biopsy argued that the additional information could benefit the patients through guidance in decisions about adjuvant chemotherapy. In case of approximately one-half of patients in whom there was residual nodal disease, it was also argued that complete ALND could influence survival via local-regional control of the axilla. The therapeutic benefit of complete ALND was minimal.^[1]^

Axillary web syndrome has been common after the axillary surgery, frequently affecting the breast cancer patients. Under this condition, patients develop one or more linear bands of firm tissue, also known as “cords,” in the axilla and the arm, associated with pain and limited range of motion of the shoulder and the arm.^[2]^ Although the standard of care for breast cancer patients with SLN metastases depends upon the performance of complete ALND, many questions need to be answered for a complete ALND in every patient with detectable SLN metastases, particularly those in whom the perceived risk of additional disease was low.[^3^,^4^] The SLN biopsy alone, without complete ALND, has been adopted at many institutions as an accurate method of staging the axilla while avoiding much of the morbidity associated with a complete ALND. Approximately 50% of patients with positive SLNs were found to have no other nodal metastases.^[5]^

Normally, clinical prediction models combine multiple predictors to provide an insight into the relative effects of the predictors in the models. Prediction of the status of non-SLN metastases becomes more and more crucial in the current era of personalized medicine in breast cancer patient. Developments in imaging, biomarkers, and “omics” research led to many new predictors for diagnosis and prognosis. A more user-friendly nomogram for predicting the status of the non-SLN metastases could help breast cancer patients to avoid many complications mentioned thereupon.

This study was intended to develop a nomogram that would allow greater individualization of a patient's risk of non-SLN metastases estimates by simultaneously taking into account several pertinent characteristics specific to the patient. With a more precise and individualized estimation, both the physician and the patient would be better able to weigh the pros and cons of any further axillary dissection and to avoid an unnecessary ALND.

## 2 Materials and methods

### 2.1 Patients

The data were provided by the Chinese Society of Clinical Oncology (CSCO) Breast Cancer Database collaborated Group (Research number is CSCO BC RWS 18005). Between January 1, 2010, and September 30, 2017, 50,000 breast cancer patients were included. There were 1036 women with breast cancer with a positive SLNB who underwent completed ALND in an ongoing SLN program. All the patients had primary invasive breast carcinoma with clinically negative axilla and no prior systemic treatment, with a successful SLN biopsy in which the metastatic disease was identified. There were 470 patients who fulfilled the age, tumor size, pathological type, nuclear grade, lymphovascular invasion, estrogen receptor status, progesterone receptor status, Her-2 status, Ki-67 count, menopause status, and number of total SLNs, and the number of positive SLNs.

### 2.2 Histopathological evaluation

The Formain-fixed, paraffin-embedded specimens from 470 patients were retrieved and reassessed by examining the Hematoxylin and eosin-stained histologic sections. The histologic type of all the specimens was reconfirmed in consonance with the breast invasive ductal carcinoma, defined according to the World Health Organization classification. Histologic grading was carried out using the Nottingham-combined histologic grade (Elston-Ellis modification of Scarff-Bloom-Richardson grading system). The pathologic TNM stage was judged by the 7th American Joint Committee on Cancer.^[6]^ Analyses for estrogen receptor (ER), progesterone receptor (PR), HER2, were conducted conforming to the recommended guidelines of the American Society of Clinical Oncology and College of American Pathologists.[^7^,^8^] The menopause status were conducted conforming to the recommended Collaborative Group on Hormonal Factors in Breast Cancer.^[9]^

The variables used included age, tumor size, pathological type, nuclear grade, lymphovascular invasion, estrogen receptor status, progesterone receptor status, Her-2 status, Ki-67 count, menopause status, number of total SLNs, and the number of positive SLNs.

### 2.3 Statistical analysis

All the 470 patients included were randomly split into training group for development of the prognostic model and validation group only for model testing purposes, according to 7 to 3. Continuous variables were expressed as mean (SD), and categorical variables were expressed as number and proportion as appropriate. Comparisons of clinical characteristics between groups were performed by using Student *t* test or the Wilcoxon rank-sum test for continuous variables and the chi-squared test or Fisher exact test for categorical variables, where deemed appropriate. In the training cohort, univariable logistic regression analysis quantified the association between each individual predictor variable and non-SLN metastases. Multiple logistic regression was performed using all selected predictor variables to determine the independent prognostic risks. Contribution of each predictor variable within the final model was presented as *β* coefficients and odds ratios (ORs) with 95% confidence intervals (CI).

Two prognostic models and corresponding nomogram were constructed. The model 1 was based on the variables associated with non-SLN metastases at a significant level in multivariate analysis. Model 2, including the variables in model 1, added several variables on the basis of clinical plausibility. ROC analysis was performed to calculate the area under curve (AUC) into evaluating the discrimination of the 2 models. The decision curves of the 2 models were also plotted to assess the benefits of nomogram-assisted decisions in a clinical context. Furthermore the comparison between the 2 models adopted Delong method. ROC curves and 95% CI of final model were estimated using bootstrap resampling (times = 500) to decrease the overfit bias.

Performance testing of the final model was assessed in training and validation group in terms of discrimination and calibration. Calibration was assessed graphically using the observed outcome plotted against the predicted probability of the outcome.

Statistical analyses were carried out with Empower (R) (http://www.empowerstats.com, X&Ysolutions, Inc., Boston, MA) and R (http://www.R-project.org). *P* < .05 was considered as a statistically significant difference.

## 3 Results

### 3.1 Clinicopathologic characteristics

A total of 470 patients who met the inclusion criteria were enrolled, and 3 (347 cases):1 (123 cases) patients were divided into the training and validation cohorts, respectively.

The clinicopathologic characteristics of the patients have been listed in Table 1. The baseline clinicopathologic data were similar between the training and validation cohorts. The positive status of SLNs was found in 125 (36.02%) and 43 (34.96%) patients in the 2 cohorts, respectively.

**Table 1:**
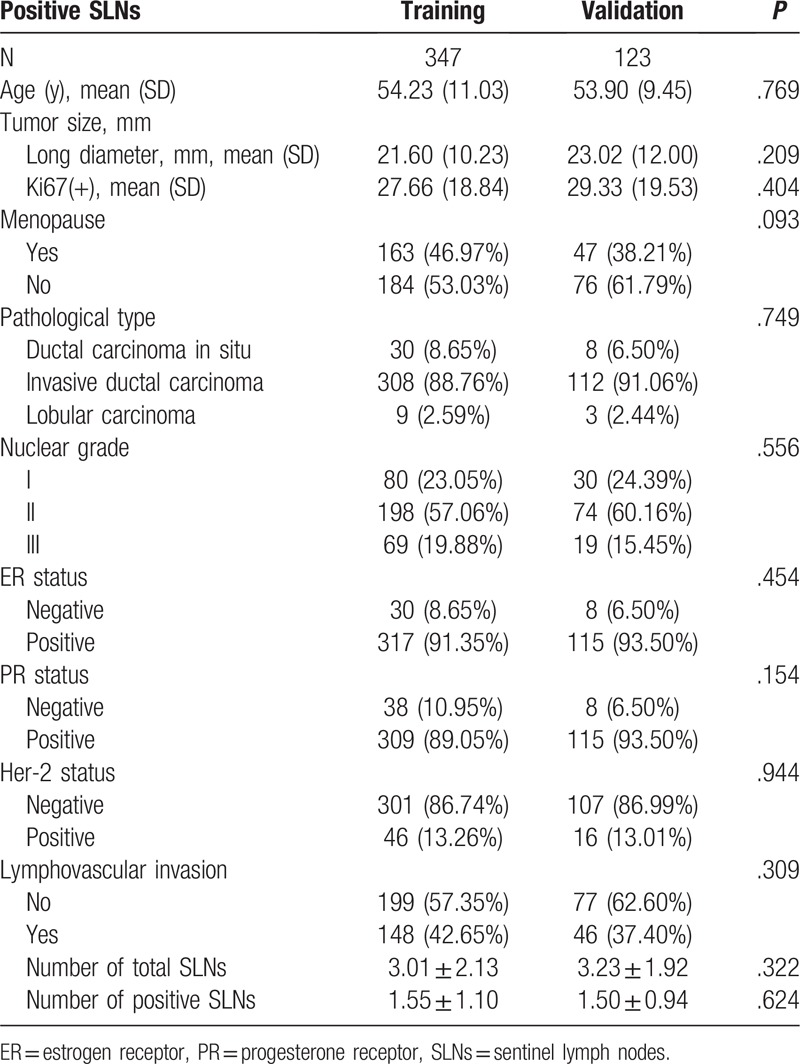
Descriptive characteristics of the patient population.

All variables listed under Table 1 were used for the univariate and multivariate Cox regression analysis. The results of the univariate logistic analysis were presented in Table 2.

**Table 2:**
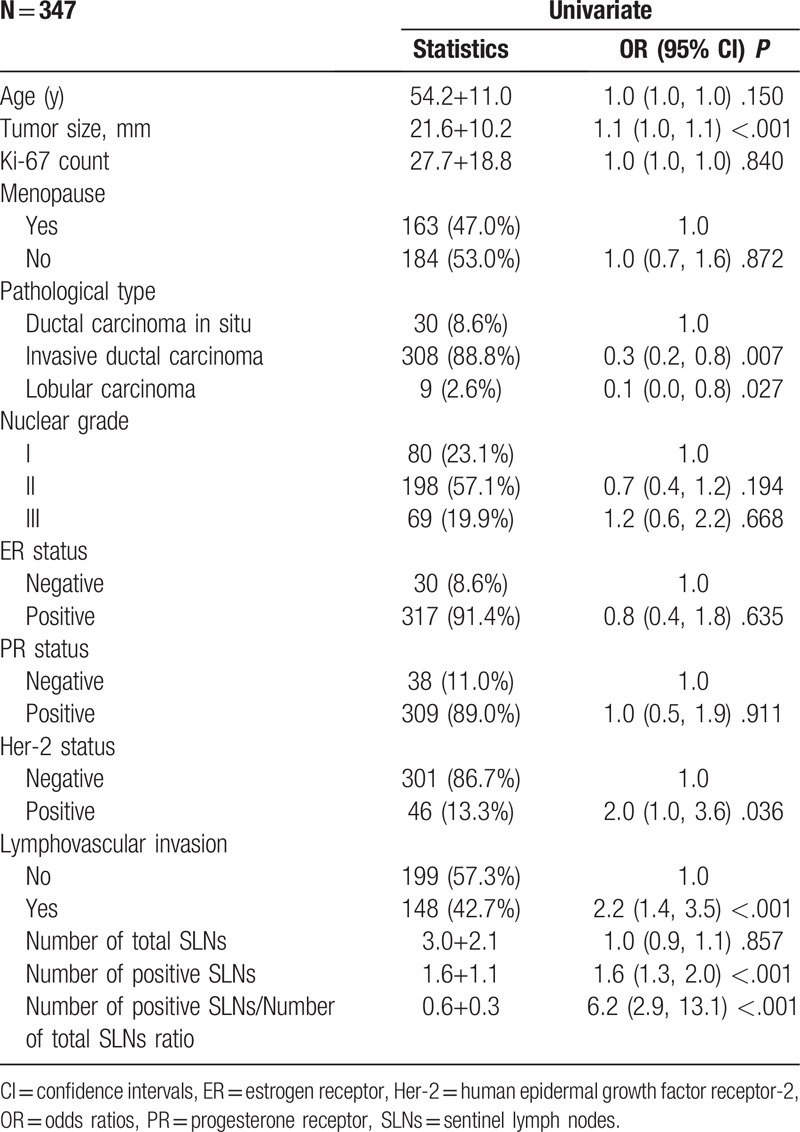
Univariable associations between the clinical variables and non-sentinel lymph node metastases in the training group.

### 3.2 The 2 predictive model 1 and model 2

**Figure 1:**
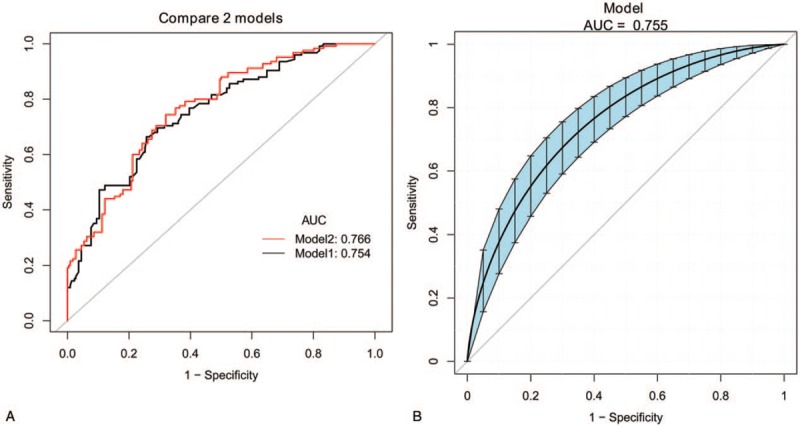
The compare of the 2 models about ROC curve (A) and 95% CI of model 1 (B). CI = confidence interval, ROC = receiver operating characteristic.

**Figure 2:**
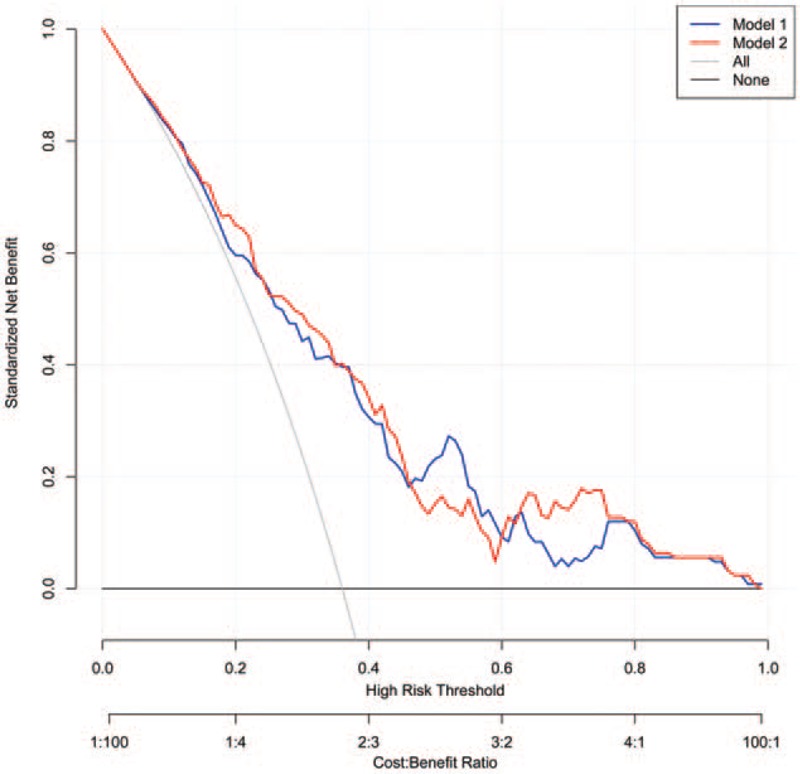
Decision curve analysis for the SLN metastases evaluation of the final feature set. The gray line is the decision curve for treat all patients SLN metastases status as positive. The dark line is the decision curve for non-SLN metastases as negative. The *y*-axis measures the net benefit. The blue and red lines represent the model 1 and model 2, respectively. Cost:Benefit Ratio stand for positive:negative ratio. SLN = sentinel lymph node.

**Figure 3:**
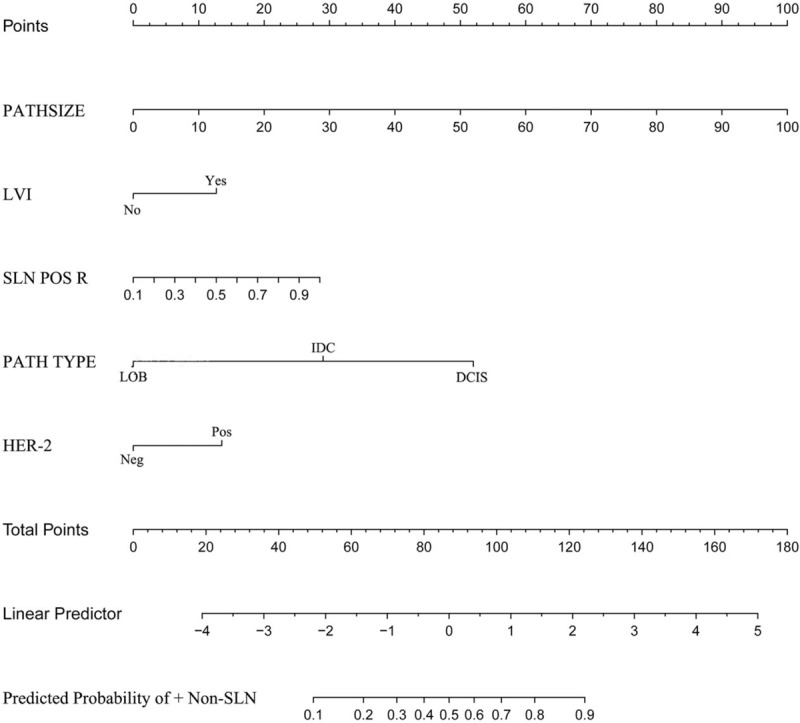
Nomogram to predict likelihood of additional, non-sentinel lymph node (non-SLN) metastases in a patient with a positive SLN. PATHSIZE: pathological size, PATH TYPE: pathological type, LVI: lymphovascular invasion, SLN POS R: the number of positive SLNs/number of total SLNs ratio and Her-2 status. Rows 2–6 represent the variables included in the model. For an individual patient, each variable is assigned a point value (uppermost scale, Points) based on the histopathological characteristics. A vertical line is made between the appropriate variable value and the points line. The assigned points for all 5 variables are summed, and the total is found in row 7 (Total Points). Once the total is located, a vertical line is made between Total Points and the final row, Row 8 (Predicted Probability of + non-SLN).

**Figure 4:**
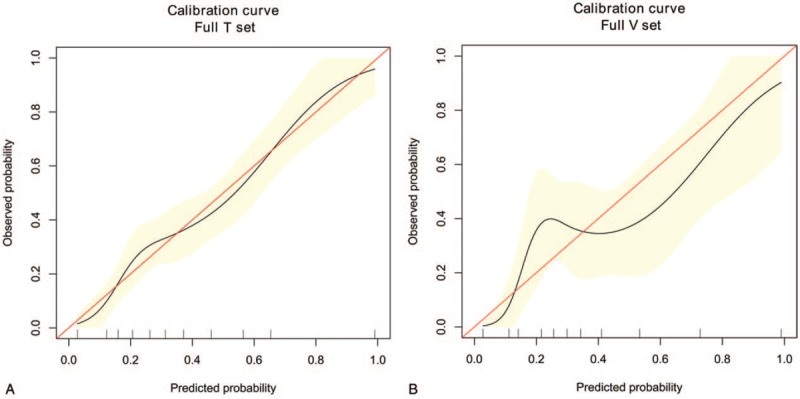
Validity of the predictive performance of the nomogram in estimating the risk of non-SLN metastases presence in the training cohort (full T set) (n = 347) (A). Validity of the predictive performance of the nomogram in estimating the risk of non-SLN metastases presence in the validation cohort (full V set) (n = 123) (B). non-SLN = non-sentinel lymph node.

In the multivariable logistic regression analysis, pathological size of the tumor, pathological type, lymphovascular invasion, the number of positive SLNs/number of total SLNs ratio and Her-2 status (*P* < .05 for each) were determined (Table 3). Hence, the first model was developed with the variables. The area under ROC curve was 0.754. As age, estrogen receptor status, progesterone receptor status, Ki-67 count, and the menopause status are important for prognosis with breast cancer, the second model was developed with age, pathological size of the tumor, pathological type, lymphovascular invasion, the number of positive SLNs/number of total SLNs ratio, Her-2 status, estrogen receptor status, progesterone receptor status, Ki-67 count, and the menopause status. The area under the ROC curve was 0.766. No difference was observed between model 1 and model 2 under the ROC curve *P* = .243 (Table 4) (Fig. 1). Both the models showed good decision curves (Fig. 2). The model 1 was selected as the training nomogram. The independently associated risk factors were used to form the non-SLN metastases risk estimation nomogram (Fig. 3). The resultant model was internally validated using the bootstrap validation method. In the validation cohort, the nomogram displayed AUC of 0.703 (95% CI, 0.609–0.796) in the estimation of non-SLN metastases risks. Both training and validation cohorts indicated good calibration curves in the risk estimations (Fig. 4).

**Table 3:**
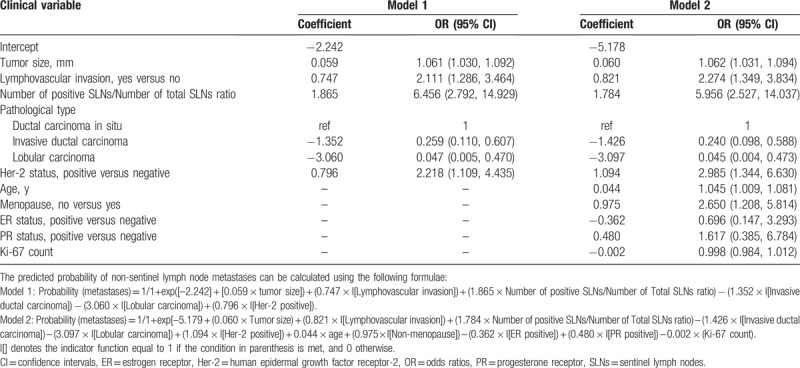
Multivariable associations between the clinical variables and non-sentinel lymph node metastases for model 1 and model 2.

**Table 4:**
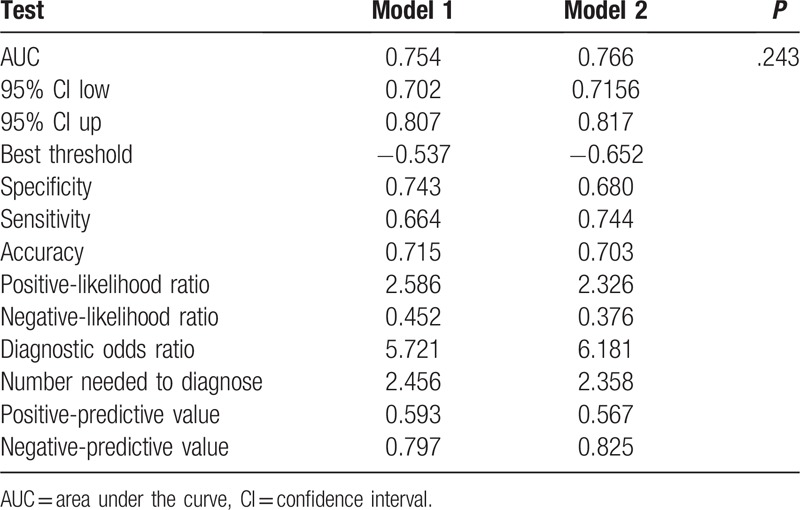
Compare 2 predictive models of AUC.

The positive and negative predictive values of the nomograms were calculated, indicating positive predictive values of 59.3% and 48.6% and negative predictive values of 79.7% and 83.0% for the training and validation cohorts, respectively (Table 5).

**Table 5:**
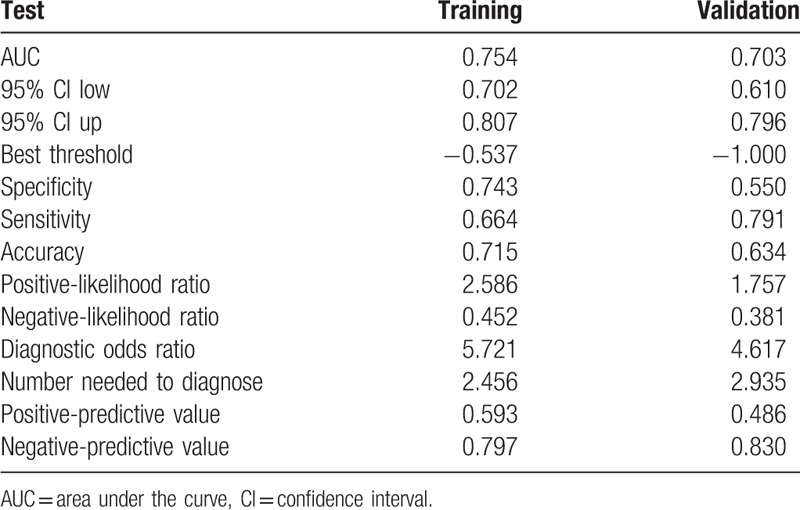
Descriptive characteristics of the training and validation group.

## 4 Discussion

The ALND furnishes an accurate and complete staging of the axilla, aids in decision making regarding adjuvant chemotherapy and radiotherapy, and is thought to have advantages in reducing the local recurrence rate as well as survival advantages^[10]^ and a significant number of patients with positive SLNs do not have any additional metastases in non-sentinel lymph nodes (NSLNs). Nevertheless, ALND carries a significant risk of morbidity. It is estimated that around 15% to 30% of the patients develop permanent lymphedema. Other complications, such as wound infection, seroma, arm weakness, decreased range of shoulder movement, and neurologic changes have also been documented.[^11^,^12^,^13^] Hence, the therapeutic benefits of ALND in patients with tumor positive SLNs have been questioned. A prolonged follow-up of 25 to 30 years did not find any survival advantage in the patients.^[14]^ Another study also found no evidence of axillary recurrence in a group of low-risk patients who did not have further ALND.^[15]^

The MSKCC (Memorial Sloan Kettering Cancer Center), Cambridge,^[12]^ Turkish,^[16]^ Stanford,^[17]^ MDACC^[18]^ (University of Texas MD Anderson Cancer Center), Tenon,^[19]^ and MOU^[20]^ (Masarykuv Onkologický Ustav, Masaryk Memorial Cancer Institute) models were used to predict the probability of additional axillary nodal metastases after positive sentinel lymph node biopsy, but they are not for Chinese population. So we proceeded with 2 logistic models to predict the probability of further additional non-SLNs metastases to independently validate with variable results in breast cancer. A model with an AUC of 0.50 was equivalent to the toss of a coin. A model with an AUC of 0.70 to 0.80 was considered good. Whereas, one with an AUC of 0.80 to 0.90 had excellent discrimination.[^21^,^22^] Model 1 was created with pathological size of the tumor, pathological type, lymphovascular invasion, the number of positive SLNs/number of total SLNs ratio, Her-2 status based on multivariable logistic regression (*P* < .05 Table 2) to predict the presence of additional disease in the non-SLNs of these patients. Age, estrogen receptor status, progesterone receptor status, Ki-67 count, menopause status^[23]^ are important to breast cancer patients for prognosis and treatment, so we created model 2 with age, estrogen receptor status, progesterone receptor status, Ki-67 count, menopause status, being based on the clinicopathologic variables that were important for the overall survival rate and disease-free survival rate, besides the variables in model 1. The AUC of the 2 models were 0.754 and 0.766, respectively, indicating that the nomgrams were good and there was no difference between model 1 and model 2 with respect to the AUC, *P* = .243. The number of variables in model 1 was less than model 2. Of the currently available prediction tools, a nomogram is known to have high accuracy and good discrimination characteristics in predicting outcomes with ease of use.^[24]^ Hence, model 1 was considered to be more convenient and easier to collect data. Model 1 was created with size of the tumor, pathological type, lymphovascular invasion, and the number of positive SLNs/number of total SLNs ratio, those are known within 30 minutes after the tumor and the SLN resection. The nodal and tumor tissue was quick frozen in liquid nitrogen, and a single 5 μm thick section stained with hematoxylin and eosin (H&E) was examined intraoperatively (frozen-section analysis). The size of the tumor, pathological type, lymphovascular invasion, and the number of positive SLNs/number of total SLNs ratio is known after the frozen-section analysis. So our nomograms utilize readily available clinical information and allow quick calculation, a complete ALND was done immediately.

Model 1 was a more user-friendly nomogram than model 2. In our study estrogen receptor status, progesterone receptor status, menopause status, and the Ki-67 count did not play a significant role in predicting the metastases of ALND. We had similar results with MSKCC.^[4]^

The model 1 was selected as the training cohort (n = 347), while a prospective study further confirmed the reliability of the nomogram (n = 123). In the validation cohort, the nomogram displayed a C-index of 0.703 (95% CI, 0.609–0.796) during the estimation of non-SLN metastases risk. The positive and negative predictive values of the nomogram were determined, resulting in positive predictive values of 59.3% and 48.6% and negative predictive values of 79.7% and 83.0% respectively, for the training and validation cohorts. The optimal calibration curves demonstrated the consonance between the predictions and the actual observations. Calibration plots graphically showed good agreement on the presence of non-SLN metastases between the risk estimation by the nomogram and histopathologic confirmation on surgical specimens. There was also a good calibration curve in case of the risk estimation.

We could use the model 1 to predict the probability of further additional non-SLNs metastases. Our nomogram suggests that if a patient has a 10% risk of having non-SLN metastases. Should she undergo completion ALND? Given this scenario, some will judge that a 15% risk of additional, non-SLN metastases justifies further ALND; others will not. The nomogram itself makes no actual treatment recommendations.

Nonetheless, there were several limitations to our model. First, the number of patients was small. Second, a prospective study was needed to further confirm the reliability of the nomograms. Finally, since the model was based on clinicopathologic data, specific markers to estimate the non-SLN metastases could have further improved the accuracy.

## 5 Conclusion

With the important clinical question of whether to perform a complete ALND in a patient with a positive SLN biopsy arising more and more frequently, the model 1 was able to provide an easy-to-use tool with which to simultaneously incorporate several important variables into the estimation of the risk of additional, non-SLN metastases.

## Author contributions

**Conceptualization:** Yunjiang Liu.

**Data curation:** Shuo Zhang, Feng Liu.

**Formal analysis:** Feng Liu.

**Investigation:** Yaheng Zhao.

**Methodology:** Lihua Zheng, Feng Liu.

**Visualization:** Feng Liu.

**Project administration:** Yunjiang Liu.

**Resources:** Shuo Zhang.

**Writing – original draft:** Lihua Zheng

**Writing – review & editing:** Lihua Zheng, Feng Liu, Yunjiang Liu.
